# Acupuncture for behavioral and psychological symptoms of dementia: A protocol of systematic review

**DOI:** 10.1097/MD.0000000000024341

**Published:** 2021-02-12

**Authors:** Chan-Young Kwon, Boram Lee

**Affiliations:** a Department of Oriental Neuropsychiatry, Dong-eui University College of Korean Medicine, Busanjin-gu, Busan; b Clinical Medicine Division, Korea Institute of Oriental Medicine, Yuseong-gu, Daejeon, Republic of Korea.

**Keywords:** acupuncture, behavioral and psychological symptoms of dementia, dementia, East Asian traditional medicine, protocol, systematic review

## Abstract

**Background:**

Dementia is a major mental health problem worldwide, and an optimal anti-dementia drug that could modify its core symptoms has not been developed yet. Behavioral and psychological symptoms of dementia (BPSD), an important clinical manifestation of dementia, is closely related to disease burden, caregiver burden, and consequent social burden. In general, many experts and international guidelines prefer non-pharmacological interventions, including psychosocial intervention, and complementary and integrative medicine in the management of BPSD. However, in clinical settings, psychotropic drugs are frequently used; therefore, the need to establish and actively use effective non-pharmacological interventions is emphasized. Therefore, in this systematic review, we will evaluate the effectiveness and safety of acupuncture, a promising non-pharmacological complementary and integrative medicine, for BPSD.

**Methods and analysis:**

A comprehensive search will be conducted in 13 electronic medical databases. Regardless of its design, original clinical studies, such as randomized controlled clinical trials, nonrandomized controlled clinical trials, and before-after studies, will be included to assess the beneficial effects and safety of acupuncture on BPSD. The severity of BPSD symptoms assessed by the validated tool will be considered as a primary outcome. The secondary outcome included the total effective rate, daily living activities and quality of life of patients, burden and quality of life of caregiver, placement in a long-term care facility from home, and safety data. The study selection, data extraction, and quality assessment process were performed by 2 independent researchers. The methodological quality of the included studies will be assessed using validated tools according to its design, such as the Cochrane Collaboration's risk of bias tool. RevMan version 5.3 will be used to perform the meta-analysis, with mean differences for continuous outcomes and risk ratio for binary outcomes, and 95% confidence intervals. A fixed- or random-effects model will be used according to the heterogeneity and number of included studies.

**Ethics and dissemination:**

As this protocol is for a systematic review, ethical approval is not required. The results of the systematic review will be disseminated by the publication of a manuscript in a peer-reviewed journal or presentation at a relevant conference.

**Registration number:**

OSF (URL: https://osf.io/hu5ac), PROSPERO (CRD42020211005) (URL: https://www.crd.york.ac.uk/prospero/display_record.php?ID=CRD42020211005).

## Abstract 2

Supplemental Digital Content is available in the text

## 1 Introduction

Dementia is related to neurodegenerative diseases caused by various causes and cognitive impairment, including memory loss, making it impossible to maintain a normal level of daily life, and is regarded as an increasingly serious global public health problem.^[1]^ The prevalence of this debilitating disease is rapidly increasing, and is expected to reach 81.1 million by 2040.^[2]^ Among the diseases that cause dementia, the most common are Alzheimer disease (AD) and vascular dementia. However, in addition to this, other diseases, including Parkinson disease, Lewy body disease, and Huntington disease, can also cause dementia. The clinical symptoms of dementia can be largely divided into core symptoms and behavioral and psychological symptoms of dementia (BPSD).^[3]^ Among them, cognitive symptoms, which are the core symptoms of dementia, include memory loss, impaired calculation ability, and impaired spatial perception. However, the optimal anti-dementia drug that modifies the core symptoms of dementia has repeatedly failed to develop.^[4]^ Currently, only 4 drugs, including 3 cholinesterase inhibitors and memantine, are approved for the treatment of dementia by the U.S. Food and Drug Administration, and they only have the effect of short-term symptom relief and delay disease progression.^[4]^

The other important symptom groups of dementia are non-cognitive symptoms or BPSD, which include depression, aggression, impulsiveness, anxiety, agitation, paranoia, delusion, hallucination, and wandering,^[3]^ and are considered major causes of disease burden, caregiver burden, and consequent social burden.^[5–7]^ According to epidemiological studies, BPSD is present in almost all dementia patients, and its impact on their families and society are profound.^[8]^ In general, many experts and international guidelines prefer non-pharmacological interventions, including psychosocial intervention, complementary, and integrative medicine, in the management of BPSD.^[9–13]^ However, in clinical settings, psychotropic drugs are frequently used,^[14]^ so the need to establish and actively use effective non-pharmacological interventions is emphasized.

East Asian traditional medicine is a valuable source of medicine in Asian countries. Today, acupuncture and some herbal medicines are used worldwide in various medical fields, including neurodegenerative diseases. Although acupuncture tends to be the most well-known in the field of pain medicine,^[15]^ some researchers have suggested its use in dementia for both BPSD and its core symptoms. For example, a systematic review in 2015 that analyzed the effectiveness and safety of acupuncture for AD patients reported that the acupuncture group showed better results than the medication group in terms of cognitive function and activities of daily living (ADL).^[16]^ Acupuncture also can be used for BPSD because preclinical and clinical evidence is accumulating that this treatment has the effect of improving psychological symptoms, such as depression, impulsiveness, anxiety, agitation, and sleep disturbances.^[17–19]^ Moreover, acupuncture is a potentially effective, complementary, and integrative medicine approach used to manage late-life mood and cognitive disorders, but the strength of the evidence should be further strengthened.^[20]^

Therefore, synthesizing the evidence for the effectiveness and safety of acupuncture for BPSD may help establish an effective non-pharmacological strategy for dementia management in terms of evidence-based medicine. This work can potentially further help reduce the burden of diseases, caregivers, and social burdens caused by dementia. The research question of this systematic review is “could acupuncture be effective and safe for BPSD?” Thus, the purpose of this systematic review was to evaluate the effectiveness and safety of acupuncture for BPSD, regardless of the dementia type or severity.

## 2 Methods

### 2.1 Study registration

The systematic review protocol is registered in the OSF registries (URL: https://osf.io/hu5ac) and the International Prospective Register of Systematic Reviews (PROSPERO) (registration number, CRD42020211005) (URL: https://www.crd.york.ac.uk/prospero/display_record.php?ID=CRD42020211005). If protocol amendments occur, the dates, changes, and rationales will be tracked in PROSPERO. This protocol was reported in accordance with the Preferred Reporting Items for Systematic Review and Meta-Analysis Protocols 2015 statement (Supplemental Digital Content 1; http://links.lww.com/MD/F558).^[21]^

### 2.2 Data sources and search strategy

**Figure 1:**
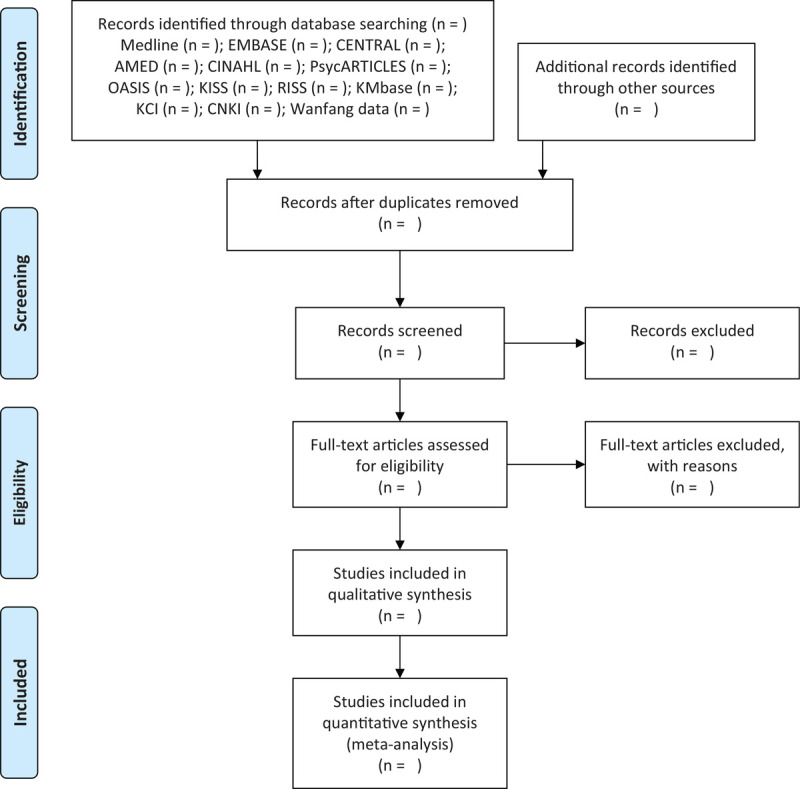
A PRISMA flow diagram of the literature screening and selection processes. AMED = allied and complementary medicine database; CENTRAL = Cochrane Central Register of Controlled Trials; CINAHL = cumulative index to nursing and allied health literature; CNKI = China National Knowledge Infrastructure; KCI = Korea Citation Index; KISS = Korean Studies Information Service System; KMbase = Korean Medical Database; OASIS = Oriental Medicine Advanced Searching Integrated System; RISS = Research Information Service System.

Two independent researchers (CY Kwon and B Lee) will search the following electronic bibliographic databases from their inception dates to November 2020: 6 English databases (MEDLINE via PubMed, EMBASE via Elsevier, the Cochrane Central Register of Controlled Trials, Allied and Complementary Medicine Database via EBSCO, Cumulative Index to Nursing and Allied Health Literature via EBSCO, and PsycARTICLES via ProQuest), 5 Korean databases (Oriental Medicine Advanced Searching Integrated System, Korean Studies Information Service System, Research Information Service System, Korean Medical Database, Korea Citation Index), and 2 Chinese databases (China National Knowledge Infrastructure and Wanfang Data). Also, we will search the reference lists of the relevant articles and will manually search Google Scholar to identify additional gray literature for inclusion (Fig. 1). Table 1 shows the search strategy in Medline via PubMed.

**Table 1:** Search strategies for the Medline via PubMed.

#1. Dementia[MeSH] OR dement^∗^[Title/Abstract] OR Alzheimer^∗^[Title/Abstract] OR “Lewy body”[Title/Abstract] OR Huntington^∗^[Title/Abstract] OR Parkinson^∗^[Title/Abstract] OR “Pick disease”[Title/Abstract] OR “cognitive impairment”[Title/Abstract]
#2. Acupuncture[MeSH] OR Electoacupuncture[MeSH] OR “Acupuncture Therapy”[MeSH] OR “Acupuncture Points”[MeSH] OR acupunct^∗^[Title/Abstract] OR electroacupunct^∗^[Title/Abstract] or electro-acupunct^∗^[Title/Abstract] OR acupoint[Title/Abstract]
#3. #1 AND #2

### 2.3 Inclusion criteria

#### 2.3.1 Types of studies

Original clinical studies, including randomized controlled clinical trials (RCTs), nonrandomized controlled clinical trials, and before-after studies to assess the beneficial effects and safety of acupuncture on BPSD were included. There was no restriction on publication language or publication status.

#### 2.3.2 Types of participants

Studies involved people with any type of dementia in long-term care facilities, community, or specialized geriatric assessment and psychiatric units. The diagnostic criteria will allow the standardized diagnostic criteria in the Diagnostic and Statistical Manual of Mental Disorders, the International Classification of Diseases, the National Institute of Neurological and Communicative Disorders, and Stroke and the Alzheimer Disease and Related Disorders Association or other recommended diagnostic criteria. There were no restrictions on the gender, age, or race of the participants. However, studies that did not provide diagnostic criteria, or a validated tool for inclusion, studies on patients with drug allergies, or other serious illnesses such as cancer, liver disease, or kidney disease were excluded.

#### 2.3.3 Types of interventions

Studies involving any type of acupuncture (ie, manual acupuncture, electroacupuncture, auriculotherapy, etc) as monotherapy or adjunctive therapies to psychotropic drugs, such as anxiolytics, antidepressants, and antipsychotics, with or without routine care for dementia as experimental interventions will be included. For control intervention, studies involving waitlist, placebo (sham-acupuncture), or psychotropic drugs, with or without routine care for dementia will be included. Studies that do not list the details such as treatment period, treatment points (ie, acupoints), and stimulation methods of acupuncture performed will be excluded. A study involving psychotherapy, which is not an intervention of interest in this review, as experimental or control interventions will also be excluded.

#### 2.3.4 Types of outcome measures

The primary outcome measures are the severity of BPSD symptoms, such as Behavior Pathology in Alzheimer Disease Rating Scale,^[22]^ Neuropsychiatric Inventory,^[23]^ Cohen-Mansfield Agitation Inventory,^[24]^ and Brief Psychiatric Rating Scale.^[25]^ The secondary outcome measures include

(1) total effective rate for BPSD symptoms;
(2) ADL of patients such as Barthel Index,^[26]^ Katz Index,^[27]^ and the Functional Independence Measure,^[28]^ as well as instrumental ADL such as Activities of Daily Living Prevention Instrument,^[29]^ Alzheimer Disease Activities of Daily Living International Scale,^[30]^ and Bayer Activities of Daily Living Scale^[31]^;
(3) quality of life of patients such as Alzheimer Disease Related Quality of Life,^[32]^ Dementia Quality of Life Instrument,^[33]^ and Quality of Life in Late-Stage Dementia Scale^[34]^;
(4) caregiver burden of caregiver such as Caregiver Burden Inventory^[35]^;
(5) quality of life of caregiver such as Short Form 36 Health Survey^[36]^;
(6) placement in long term care facility from home; and
(7) safety data such as incidence of adverse events (AEs) and treatment discontinuation due to total or serious AEs.

### 2.4 Study selection

First, 2 researchers (CY Kwon and B Lee) will screen independently to identify titles and/or abstracts of studies that potentially meet the inclusion criteria. Second, those 2 researchers will independently assess the full texts of these potentially eligible studies for eligibility. Any disagreements between the researcher will be resolved through discussion between them. EndNote X8 (Clarivate Analytics, Philadelphia, PA) will be used to manage quotations of included articles.

### 2.5 Data extraction

A standardized, pre-defined, pilot-tested form will then be used to extract data from the included studies for assessment of study quality and evidence synthesis. The extracted information will include the first author's name, year of publication, country, sample size and dropout, details of participants, experimental intervention, comparison, duration of intervention, main outcome measures, AEs, and information for assessment of the risk of bias (RoB). Two researchers (CY Kwon and B Lee) will extract the data independently, and any discrepancies will be identified and resolved through discussion (with other researchers, where necessary). Excel 2016 (Microsoft, Redmond, WA) and Dropbox (Dropbox, Inc.San Francisco, California, USA) folders were used to perform the data extraction process and to share the extracted data, respectively. When the data are insufficient, ambiguous, or missing, we will contact the corresponding authors of the original studies via e-mail.

### 2.6 Quality assessment

For included RCTs, the Cochrane Collaboration's RoB tool will be used to assess the RoB. By using the tool, 7 domains of each RCT, including random sequence generation, allocation concealment, blinding of participants, personnel, and outcome assessors, completeness of outcome data, selective reporting, and other biases will be assessed as “low risk,” “unclear risk,” or “high risk.”^[37]^ In case of other bias, the statistical baseline imbalance severity between the treatment and control groups, including the participant's mean age, sex, disease period, or disease severity, will be considered. For included nonrandomized controlled clinical trials, the risk of bias in non-randomized studies of interventions tool will be used to assess the RoB.^[38]^ For included before-after studies, the Quality Assessment Tool for Before-After (Pre-Post) Studies With No Control Group, proposed by the National Heart, Lung, and Blood Institute, will be used to assess the RoB.^[39]^ For case reports/case series, the Quality Assessment Tool for Case Series Studies, proposed by the National Heart, Lung, and Blood Institute, will be used to assess the RoB.^[39]^ Two researchers (CY Kwon and B Lee) will assess the quality of included studies independently, and any discrepancies will be identified and resolved through discussion (with other researchers, where necessary). Each evaluation will be recorded in an Excel 2016 (Microsoft) file and will be shared among the authors by using Dropbox (Dropbox, Inc.) folders.

### 2.7 Data synthesis and analysis

We will provide a narrative synthesis of the findings from all included studies, including the demographic characteristics of the participants, the details of the interventions, the outcomes, and the results. Studies have used the same type of interventions and comparators, with the same outcome measures; quantitative synthesis will be performed using Review Manager software, version 5.4 (Cochrane, London, UK), with mean differences for continuous outcomes and risk ratio for binary outcomes and 95% confidence intervals. Heterogeneity between the studies in terms of effect measures will be assessed using both the *χ*^2^ test and the *I*^2^ statistic, and we will consider an *I*^2^ value greater than 50% as indicative of substantial heterogeneity, and a value greater than 75% as indicative of considerable heterogeneity. The results will be pooled using a random-effects model if included studies have significant heterogeneity (an *I*-squared value more than 50%), while a fixed-effect model will be used if the heterogeneity is not significant, or if the number of studies included in the meta-analysis is very small (ie, less than 5), implying the estimate of the between-study variance will lack precision.^[40,41]^

#### 2.7.1 Subgroup analysis

If the necessary data are available, we will conduct a subgroup analysis according to the following criteria:

(1) severity of dementia,
(2) type of dementia,
(3) severity of BPSD, and
(4) treatment duration.

#### 2.7.2 Sensitivity analysis

We will perform sensitivity analyses to identify the robustness of the results of the meta-analysis by excluding

(1) studies with high RoB and
(2) outliers that are numerically distant from the rest of the data.

#### 2.7.3 Assessment of reporting biases

In addition, if sufficient studies are available (ie, more than 10), we will also assess evidence of publication bias using funnel plots.

### 2.8 Ethics and dissemination

As this protocol is for a systematic review, ethical approval is not required. The results of the systematic review will be disseminated by the publication of a manuscript in a peer-reviewed journal or presentation at a relevant conference.

## 3 Discussion

Dementia is a debilitating condition that causes significant public health problems worldwide.^[2]^ Additionally, BPSD, one of the important clinical manifestations of dementia, is closely related to disease burden, caregiver burden, and consequent social burden.^[5–7]^ Although the characteristics of BPSD may differ depending on dementia type,^[42]^ in addition to AD and vascular dementia (the most common causes of dementia), dementia caused by Parkinson disease, Lewy body disease, and Huntington disease is also associated with BPSD. Given the emphasis on the use of non-pharmacological interventions for BPSD,^[9–13]^ establishing an evidence basis for acupuncture on BPSD can help to optimize future coping strategies for dementia. However, as far as we know, there has been no attempt to systematically synthesize clinical evidence of acupuncture for BPSD in dementia patients. We believe that the findings of this systematic review will help solve the major public health problem, dementia, in the aspect of evidence-based medicine.

Supplemental Digital Content. Supplement 1. PRISMA-P 2015 Checklists, http://links.lww.com/MD/F558.

## Author contributions

**Conceptualization:** Chan-Young Kwon.

**Funding acquisition:** Chan-Young Kwon.

**Methodology:** Chan-Young Kwon, Boram Lee.

**Supervision:** Chan-Young Kwon.

**Writing – original draft:** Chan-Young Kwon.

**Writing – review and editing:** Chan-Young Kwon, Boram Lee.

## Abbreviations

Abbreviations: AD = Alzheimer disease, ADL = activities of daily living, AEs = adverse events, BPSD = behavioral and psychological symptoms of dementia, RCTs = randomized controlled clinical trials, RoB = risk of bias, VD = vascular dementia.
